# Cytogenetic Studies on Genoprotective Effect of *Rosa damascena* Mill. Hydrosol in Plant and Lymphocyte Test Systems

**DOI:** 10.3390/life13081753

**Published:** 2023-08-16

**Authors:** Svetla Gateva, Gabriele Jovtchev, Tsveta Angelova, Tsvetelina Gerasimova, Ana Dobreva, Milka Mileva

**Affiliations:** 1 Institute of Biodiversity and Ecosystem Research, Bulgarian Academy of Sciences, 2 Gagarin Str., 1113 Sofia, Bulgaria; spetkova2002@yahoo.co.uk (S.G.); gjovtchev@yahoo.de (G.J.); angelova_ts@abv.bg (T.A.); cvetij@yahoo.com (T.G.); 2 Institute for Roses and Aromatic Plants, Agricultural Academy, 49 Osvobojdenie Blvd, 6100 Kazanlak, Bulgaria; anadobreva@abv.bg; 3 The Stephan Angeloff Institute of Microbiology, Bulgarian Academy of Sciences, 26 Acad. G. Bonchev Str., 1113 Sofia, Bulgaria

**Keywords:** *R. damascena* Mill. hydrosol, anticytotoxicity, antigenotoxicity, *Hordeum vulgare* test system, in vitro human lymphocytes, chromosome aberrations, micronuclei

## Abstract

Bulgarian *Rosa damascena* Mill. is has been known since ancient times for its high-quality oil, hydrosol, and other aromatic products. Rose hydrosol has various biological activities, but no research on its anticytotoxic/antigenotoxic effects exists. This study aimed to assess its defense potential against the genotoxin N-methyl-N′-nitro-N-nitrosoguanidine and to test its cytotoxic/genotoxic activity in plant and human lymphocyte test systems. Endpoints for cytotoxicity (mitotic index and nuclear division index) and genotoxicity (chromosome aberration and micronuclei) were used. Hydrosol was applied as a single treatment in concentrations ranging from 3% to 20% (4 h) to assess its cytotoxic and genotoxic effects. Its protective potential against MNNG was tested by applying an experimental scheme involving (i) conditioning treatment with non-toxic or slightly toxic concentrations of hydrosol, followed by genotoxin challenge (50 μg/mL) with a 4 h intertreatment time and (ii) treatment with hydrosol and mutagen with no time between the treatments. Hydrosol induces low cytotoxicity and clastogenicity, demonstrating cytoprotective/genoprotective effects against the mutagen in both applied test systems. The hydrosol defense potential was expressed by a more than twofold reduction in both chromosomal aberrations and micronuclei and by enhancing the mitotic activity compared with that of the mutagen, regardless of the experimental conditions. The results are promising for further hydrosol applications in pharmaceutical and medical practice.

## 1. Introduction

There is growing interest in detecting and testing various plant products (essential oils, hydrosols, and extracts) for their biological activities. Many of these products are widely applied in medicine, pharmacy, and the food industry, as well as for household applications and in other spheres of human life. Plant products that can reduce the cytotoxic and genotoxic effects of different genotoxins are of great importance and pharmaceutical utility. Various oils, hydrosols, and extracts possess well-expressed DNA-protective potential [1,2,3,4,5,6,7,8]. However, the defense potential of some plant products has not yet been explored.

*Rosa damascena* Mill., belonging to the Rosaceae family, is an ornamental and medicinal plant well known for its high-quality oil. Its largest producers are Bulgaria, Turkey, and some countries in the Middle East. Although it is used mainly in high perfumery and cosmetics, it also finds applications in pharmacology and the food industry [9,10]. Many studies have been conducted on its biological activity. Rose essential oil exhibits analgetic, anti-inflammatory, antitussive, antibacterial, antifungal, testicular-defensive, and antioxidant effects [11,12,13,14,15,16], as well as antimutagenic effects [17].

**Figure 1:**
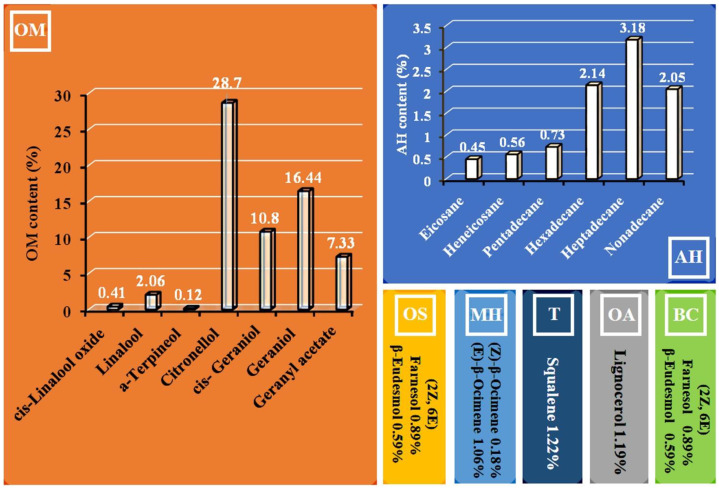
Main chemical components detected by gas chromatography/mass spectrometric (GC-MS) analysis in Bulgarian *R. damascena* Mill. hydrosol [22]. OM, oxygenated monoterpenes; BC, benzenoid compounds; AH, aliphatic hydrocarbons; OS, oxygenated sesquiterpenes; MH, monoterpene hydrocarbons; T, triterpenes; OA, oxygenated aliphatic hydrocarbons.

The steam distillation process of rose essential oil produces a hydrosol or rose water as a byproduct. Its quantity is much greater than that of rose oil itself. Hydrosol contains a variable amount of dissolved essential oil components and various secondary chemical metabolites [18]. The ratio and content of chemical compounds of rose hydrosol may also depend on the climatic and soil characteristics of growing plants and the distillation process stage [19,20]. There is quite a lot of research on the chemical composition of *R. damascena* Mill. hydrosol from different regions where this species is grown [20,21]. Our previous study detected 22 chemical compounds in Bulgarian *R. damascena* Mill. hydrosol produced in the Rose Valley, Kazanlak, Bulgaria [22] (Figure 1).

Rose hydrosol is used in cosmetics, as food flavoring [23,24], and in aromatherapy [25]. Data are also available for some of its biological activities. It can relieve gastric and duodenal spasms [23] and possesses a marked hematopoietic potential in rabbits [26]. Other authors have reported that hydrosol can ameliorate hematologic, hepatic, and renal functions and attenuate hyperglycemia in rats [27], as well as its antimicrobial effects [9,28,29]. Despite the mentioned beneficial biological activities, limited studies exist about the cytotoxic and genotoxic activities of *R. damascena* Mill. hydrosol [30], with no available research on its anticytotoxic and antigenotoxic effects. The data obtained in our previous study on the antioxidant properties of rose hydrosol [22] and the results reported in [31] with respect to the photoprotective and antioxidant activity of *R. damascena* Mill. hydrosol provoked us to test the anticytotoxic and antigenotoxic effects of rose hydrosol. Thus, we aimed to investigate the cytoprotective and genoprotective potential of *R. damascena* Mill. hydrosol against direct alkylating mutagen N-methyl-N′-nitro-N-nitrosoguanidine in two different types of test system, applying appropriate experimental schemes. Rose hydrosol was also tested for cytotoxicity and genotoxicity. For this purpose, chromosome aberrations and micronuclei were induced as sensitive and valuable tools for genotoxic screening.

## 2. Materials and Methods

### 2.1. Chemicals

RPMI 1640 medium for human lymphocyte cultivation was provided by Sigma-Aldrich, (Steinheim, Germany), fetal calf serum was provided by Sigma-Aldrich, (Sao Paulo, Brazil), and phytohemagglutinin PHA and cytochalasin B were from Sigma-Aldrich (Jerusalem, Israel). All chemicals (KCl, glacial acetic acid, colchicines, a-bromonaphthalene, pectinase, and Giemsa) used for cell fixation and staining were purchased from Sigma-Aldrich, (Steinheim, Germany). Gentamycin and 0.9% NaCl were obtained from Sopharmacy (Sofia, Bulgaria). The alkylating mutagen N-methyl-N′-nitro-N-nitrosoguanidine MNNG (CAS-Nr.: 70-25-7) used as a positive control was purchased from Fluka-AG (Buchs, Switzerland).

### 2.2. Plant Hydrosol

*R. damascena* Mill. hydrosol was prepared from rose petals (herbarium No SOM 177768) collected in the morning (6–8 a.m.) at the experimental field of the Institute for Rose and Aromatic Plants, Kazanlak, during the harvest season of 2019/2020 (May/June). Hydrosol was generated during water–steam distillation of essential oil using a semi-industrial processing line. The details for this process were previously described in [22]. The obtained rose product was stored at 4 °C in the dark in sterilized bottles for further experiments.

The quantitative and qualitative composition established by gas chromatography/mass spectrometric (GC-MS) analysis of the rose hydrosol was previously described in [22].

### 2.3. Test Systems

Two types of test system widely used in the cytogenetic screening of various natural and synthetic chemical compounds, as well as mixtures, were included in the experiments.

*Hordeum vulgare* (barley) plant test system. The structurally reconstructed karyotype MK14/2034 of *H. vulgare* characterized by seven easily distinguishable chromosome pairs was used, which allowed for investigation of the mutagen-specific features of aberration distribution patterns [32]. Despite these special properties, this karyotype is comparable in sensitivity to the standard spring barley karyotype.

In vitro human lymphocytes. Venous blood of healthy non-smoking and non-drinking donors (men and women aged 33–40 years) was used for the preparation of lymphocyte cultures [33]. The lymphocytes (1 × 10^6^ cells/mL) isolated from the whole blood were incubated at 37 °C in 3.5 mL RPMI 1640 medium with 12% fetal calf serum and 40 mg/mL gentamycin. To each culture, 0.1% mitogen-phytohemagglutinin (PHA) was added. The experiments were approved by the Commission on Ethics and Academic Unity of the Institute of Biodiversity and Ecosystem Research—BAS, Bulgaria (protocol No 1, Data: 2 March 2021).

### 2.4. Experimental Schemes

To test the cytotoxic/genotoxic activity of *R. damascena* Mill. hydrosol and to evaluate its anticytotoxic/antigenotoxic potential, different types of experimental schemes were applied. Samples with untreated cells were used as a negative control. The well-known direct alkylating mutagen MNNG (50 μg/mL) was used a positive control.

**Figure 2:**
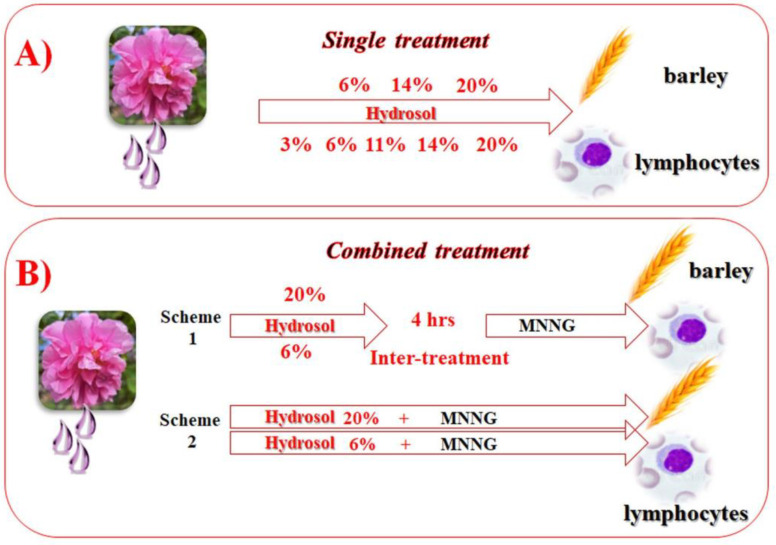
Experimental schemes of treatment: (**A**) single treatment with different concentrations of *R. damascena* hydrosol and (**B**) combined treatment with hydrosol and MNNG (Scheme 1 with 4 h intertreatment time and Scheme 2 without any time between treatments) in *H. vulgare* and human lymphocytes.

*H. vulgare*. Presoaked barley seeds (1 h tap water) were germinated for 17 h in Petri dishes on a moist filter paper at 24 °C. All subsequent procedures were performed according to the method described in [34]. To test the cytotoxic/genotoxic activity of rose hydrosol, barley root meristem cells were treated with 6%, 14%, and 20% concentrations for 4 h (Figure 2).

To assess whether the hydrosol has anticytotoxic/antigenotoxic potential, two experimental schemes with combined treatment were applied (Figure 2). When the treatment was implemented according to Scheme 1, the barley meristems were conditionally affected (60 min) with a concentration of 20% (preselected in our pilot studies), followed by a challenge with 50 μg/mL of MNNG (60 min) with a 4 h intertreatment time. When Scheme 2 was applied, the meristems were treated with 20% hydrosol (60 min), immediately followed by 50 μg/mL of MNNG (60 min), without any intertreatment time. The hydrosol concentration used as a conditioning treatment or pretreatment was chosen in our previous study as non-toxic. After each treatment, the barley root meristem cells were washed in distilled water. Recovery times of 18, 21, 24, 27, and 30 h were examined for each sample. In order to score chromosome aberrations (CAs), after the recovery times, the seedlings were affected with 0.025% colchicine in a saturated solution of a-bromonaphthalene (2 h) and fixed in a solution of ethanol: glacial acetic acid (3:1) followed by hydrolyzation in 1N HCl. Then, they were Feulgen-stained, macerated in 4% pectinase, and squashed onto slides. For micronuclei (MN) scoring, colchicine treatment was omitted, and the root tips were fixed after 30 h of recovery time [34].

Lymphocyte cultures. To test the cytotoxic/genotoxic activity of rose hydrosol by induction of chromosome aberrations, the method described in [33] was used. Five different concentrations (3%, 6%, 11%, 14%, and 20%) were applied to the lymphocytes as a single treatment for 4 h.

To evaluate the anticytotoxic/antigenotoxic potential of *R. damascena* hydrosol, a part of the lymphocyte cultures was conditionally treated (Scheme 1) with a non-toxic previously chosen concentration (6%) of hydrosol for 60 min, followed by 4 h of intertreatment time, and challenged with 50 μg/mL of MNNG (60 min). Another part of the lymphocytes (Scheme 2) was treated with 6% of rose hydrosol (60 min), immediately followed by 50 μg/mL of MNNG (60 min) without any intertreatment time. After each treatment, the cells were washed in fresh medium and incubated at 37 °C until harvest. At the 72nd h of cultivation, 0.02% colchicine was added to each sample; samples were then hypotonized in 0.56% KCl, fixed in methanol: acetic acid (3:1, *v*/*v*), and stained in a 2% Giemsa solution.

The cytokinesis-block micronucleus (CBMN) method [35] was used to assess the induction of micronuclei in binucleated lymphocyte cells. At the 44th h after PHA stimulation, cytochalasin B (6 μg/mL) was added to each lymphocyte culture. After 24 h, the cells were centrifuged, hypotonized with 0.56% KCl, and fixed in methanol: acetic acid (3:1). Post centrifugation, the cell suspension was dropped onto clean slides and stained in 2% Giemsa.

### 2.5. Endpoints

The mitotic index (MI) was calculated as an endpoint for cytotoxicity for both test systems according to following formula: MI = A/1000, where A is the number of metaphases per 1000 observed cells in each experimental variant. For human lymphocytes, the cytotoxicity was also determined by the nuclear division index (NDI) by counting 4000 cells from the experimental point using the following equation: NDI = N1 + 2N2 + 3N3 + 4N4/N, where N1–N4 corresponds to the number of cells with 1–4 nuclei, and N is the total number of cells scored.

**Figure 3:**
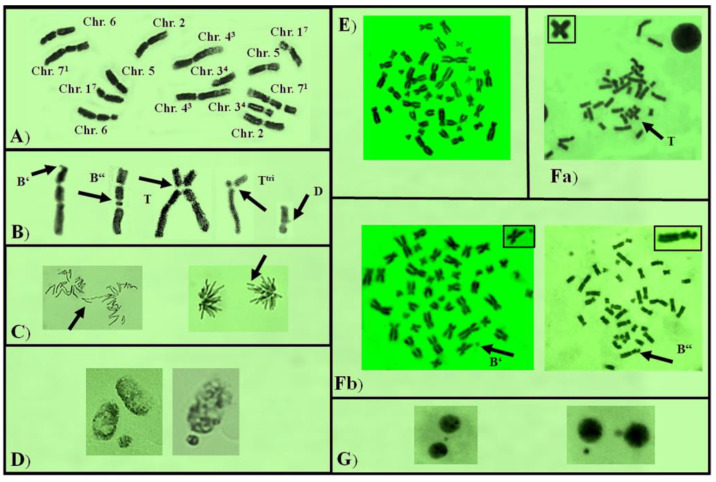
(**A**) Karyotype of *H. vulgare* reconstructed karyotype; (**B**) chromosomal aberrations: chromatid break (B′), isochromatid break (B″), translocation in NOR regions – triradial (T^trt^), and intercalary deletion (D); (**C**) mitotic disturbances; (**D**) micronuclei induced in *H. vulgare*; (**E**) karyotype of human lymphocyte; (**F**) chromosomal aberrations: (**a**) translocation (T), (**b**) chromatid break (B′), and isochromatid break (B″); (**G**) micronuclei observed in lymphocyte cells after treatment with rose hydrosol and MNNG.

As a genotoxicity end point, chromosome aberrations (CAs) were assessed as the percentage of aberrant metaphases (MwA% ± SD) and calculated for both test systems. For each experimental variant, 10,000 cells were counted. The following aberrations were determined: chromatid breaks (B′), isochromatid breaks (B″), chromatid translocations (T), and intercalary deletions (D) (Figure 3). “Aberration hot spots” were calculated in *H. vulgare* chromosomes (reconstructed barley karyotype MK14/2034) according to [36,37]. The micronuclei percentage (MN% ± SD) was calculated as another endpoint for genotoxicity based on 5000 cells for the experimental variant (Figure 3).

### 2.6. Statistical Analysis

All experiments were carried out three times. A one-way ANOVA with two-tailed Fisher’s exact test was used for statistical analysis of the different treatment variants. The following statistical differences were detected: * *p* < 0.05, significant; ** *p* < 0.01, more significant; *** *p* < 0.001, extremely significant; and *p* > 0.05, not significant.

## 3. Results

### 3.1. Cytotoxicity/Anticytotoxicity

#### 3.1.1. Cytotoxicity

**Figure 4:**
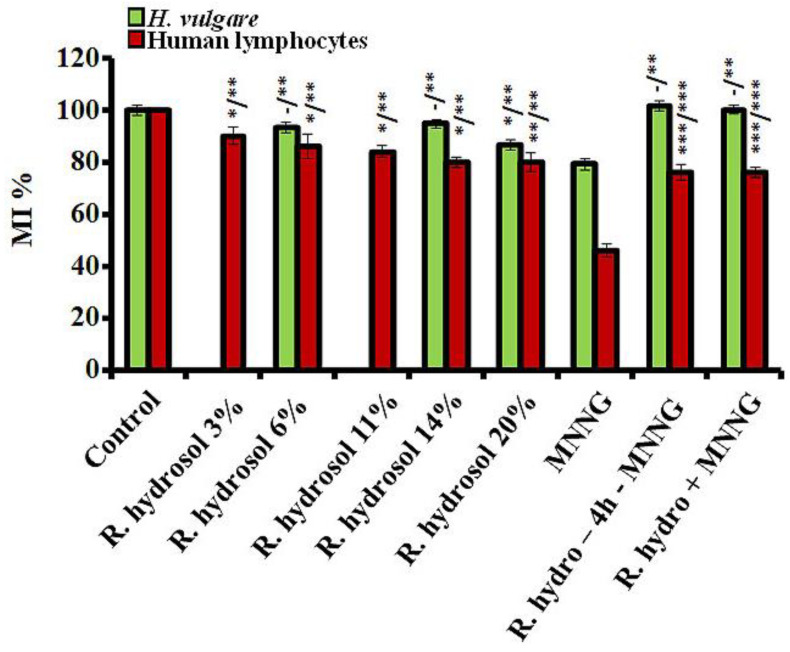
Values of MI calculated after treatment: (i) with different concentrations of *R. damascena* Mill. hydrosol; (ii) applying Schemes 1 and 2 with combined treatment with rose hydrosol and MNNG (50 μg/mL) in *H. vulgare* and human lymphocytes (- n.s, * *p* < 0.05, ** *p* < 0.01, *** *p* < 0.001; before slash: compared with the negative control: after slash: compared with MNNG).

Mitotic activity, as assessed by the values of MI in all treated variants, was calculated as a percentage of the negative control for both test systems. Treatment with the tested concentrations of rose hydrosol did not show or had a low cytotoxic effect (at 20%) in *H. vulgare* compared with the untreated meristem cells (Figure 4). Low cytotoxicity compared to the untreated control was observed in human lymphocytes (Figure 4). MI was decreased from 10% to 15% compared to untreated cells. No concentration dependence was calculated. The mitotic activity was significantly higher (*p* < 0.01, *p* < 0.001) when compared with the MNNG samples in *H. vulgare* and the cultured lymphocytes.

**Figure 5:**
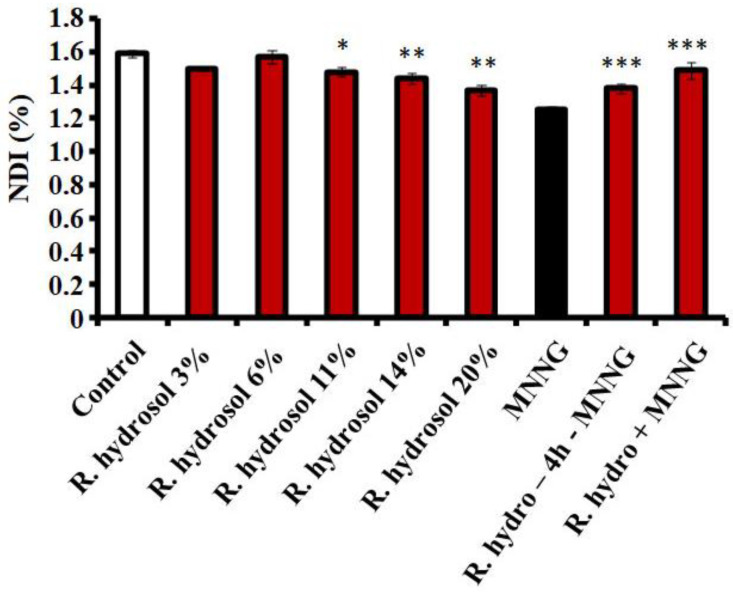
Values of NDI calculated after treatment with: (i) different concentrations of *R. damascena* Mill. Hydrosol and (ii) applying Schemes 1 and 2 with combined treatment with rose hydrosol and MNNG (50 μg/mL) in human lymphocytes, (* *p* < 0.05, ** *p* < 0.01, *** *p* < 0.001).

The cytotoxicity was assessed according to the values of NDI (the second indicator of cytotoxicity) in lymphocyte cultures, with a slight reduction (*p* < 0.05, *p* < 0.01) ob-tained after treatment with the hydrosol compared to the negative control. No concentration dependence was detected (Figure 5).

#### 3.1.2. Anticytotoxicity

The anticytotoxic effect of the rose hydrosol against MNNG was examined using the experimental designs with combined treatments (Schemes 1 and 2). Both experimental test systems reacted similarly, as assessed by the MI values, irrespective of the treatment scheme. Conditioning treatment with non-toxic or slightly toxic concentrations of *R. damascena* hydrosol prior to MNNG (50 μg/mL) significantly increased the value of MI (*p* < 0.01, *p* < 0.001) compared with that in the samples treated only with the mutagen MNNG (Figure 4). The mitotic activity increased by ~25% in barley and by 30% in human lymphocytes. A decrease in the cytotoxicity (MI) was also observed in the variants with combined treatment without any time between treatments, where the values of MI were higher (*p* < 0.01, *p* < 0.001) than those calculated in samples treated only with the mutagen (Figure 4). A well-pronounced anticytotoxic effect of rose hydrosol was obtained in lymphocyte cells by calculating NDI for both variants with combined treatments. The value of this endpoint was 1.38% ± 0.03 using Scheme 1 and 1.49% ± 0.05 using Scheme 2—both higher (*p* < 0.001) than the value calculated for variants with genotoxin only (1.25% ± 0.02) (Figure 5).

### 3.2. Genotoxicity/Antigenotoxicity

#### 3.2.1. Genotoxicity

**Figure 6:**
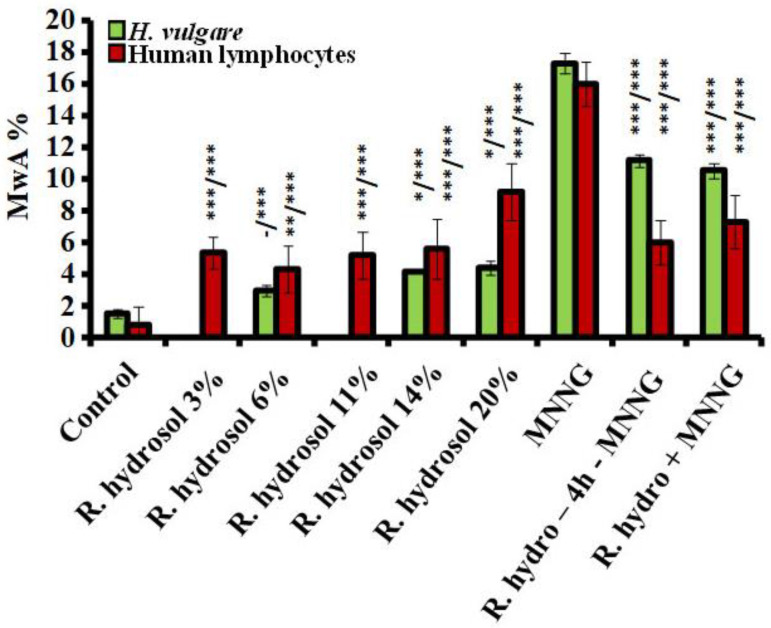
Frequency of chromosomal aberrations observed after (i) treatment with different concentrations of *R. damascena* hydrosol and (ii) applying Schemes 1 and 2 with combined treatment with hydrosol and MNNG (50 μg/mL) in *H. vulgare* and human lymphocytes (- n.s, * *p* < 0.05, ** *p* < 0.001, *** *p* < 0.001; before slash: compared with the negative control; after slash: compared with MNNG).

The tested *R. damascena* hydrosol concentrations did not show or exerted a very weak genotoxic effect in *H. vulgare* meristems compared to the untreated control assessed by induction of chromosomal aberrations (Figure 6). The induced hydrosol chromosome aberrations were in a range from 2.93% ± 0.33 for 6% to 4.40% ± 0.45 for 20%. Lymphocyte cultures were more susceptible, and after the treatment, a low but statistically significant (*p* < 0.01; *p* < 0.001) increase in the values of aberrations was observed (Figure 6). All lower tested concentrations (3–14%) induced chromosomal damages in the range from 5.33% ± 0.9 (for 3%) to 5.6% ± 1.9 (for 14%) without any concentration dependence. The highest value of aberration (20% (9.2% ± 1.7)) was reported in the sample affected by rose hydrosol in human lymphocytes. The genotoxic effect of the hydrosol was much lower (*p* < 0.001) than that of the alkylating genotoxin in both test systems (17.33% ± 0.64 in barley meristems and 16.0% ± 1.40 in lymphocyte cells) (Figure 6).

#### 3.2.2. Antigenotoxicity

Assessing the antigenotoxic potential of rose hydrosol by induction of CAs applying Scheme 1 with conditioning treatment with non-toxic or very low toxic concentrations of hydrosol, a decrease (*p* < 0.001) in the frequencies of chromosome aberrations was observed compared with that induced by direct mutagen both in barley (17.33% ± 0.64) and human lymphocytes (16.0% ± 1.4) (Figure 6). A reduction in the values of chromosome damage was also observed (*p* < 0.001) when the cells were treated using Scheme 2 (combined treatment without any intertreatment time) in both test systems. The results were more pronounced in lymphocyte cells, where chromosomal damage was decreased by more than twice using Scheme 1 (6.0% ± 1.4) and Scheme 2 (7.3% ± 1.7). In barley cells, the frequencies of the registered aberrations were 11.16% ± 0.43 and 10.53 ± 0.47, respectively. No significant difference was obtained between the values of aberrations induced after the different variants of combined treatment in both test systems. Our data obtained using CA as an endpoint for genotoxicity showed that rose hydrosol manifests a good antigenotoxic effect against the harmful action of the direct mutagen MNNG.

Rose hydrosol concentrations induced predominantly isochromatid breaks (B″), followed by a small number of intercalary deletions (D) in barley (100% B″ for 6% hydrosol; 97.22% B″ and 2.78% D for 20% hydrosol). In human lymphocytes, the spectrum of chromosomal aberrations was slightly different. Isochromatid breaks (B″) were followed by chromatid breaks (B′), as the number of the latter was higher than that of isochromatid breaks for hydrosol concentrations 11% (B″ 34.4% and B′ 66.6%) and 20% (B″ 35.0% and B′ 65.0%). After combined hydrosol and MNNG treatment in barley cells, along with the isochromatid breaks and chromatid breaks, translocations and intercalary deletions were also induced. Isochromatid breaks (B″) accounted for 91.58% and 96.20%, B′ accounted for 0.53% and 1.09%, T accounted for 4.21% and 1.62%, and D accounted for 3.68% and 1.09% applying Scheme 1 and Scheme 2, respectively. In lymphocyte cultures, only isochromatid breaks and chromatid breaks were detected.

**Figure 7:**
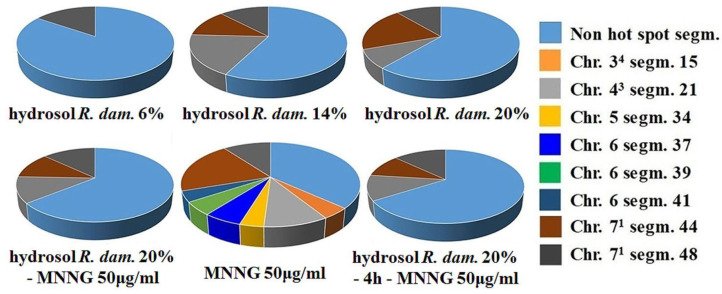
Hot spots observed in *H. vulgare* after (i) treatment with different concentrations of *R. damascena* hydrosol and (ii) applying Schemes 1 and 2 with combined treatment with *R. damascena* hydrosol and MNNG (50 μg/mL).

Interesting results were obtained for “aberrations hot spots” in barley (Figure 7). The observed aberration hot spots in the samples treated with *R. damascena* hydrosol varied from one to three, depending on the concentration applied. After the single treatment with MNNG (50 μg/mL), eight hot spots were observed. This corresponds to 36.9% hotspot-free segments or approximately 60% less than in the negative control and 20–40% less than in the hydrosol-treated variants. In two combined treatment variants (20% hydrosol—MNNG and 20% hydrosol—4 h—MNNG), three hot spots were detected for each one, which corresponds to around 35% more hotspot-free segments than in the positive control, revealing an antigenotoxic effect of rose hydrosol against MNNG. (Figure 7).

**Figure 8:**
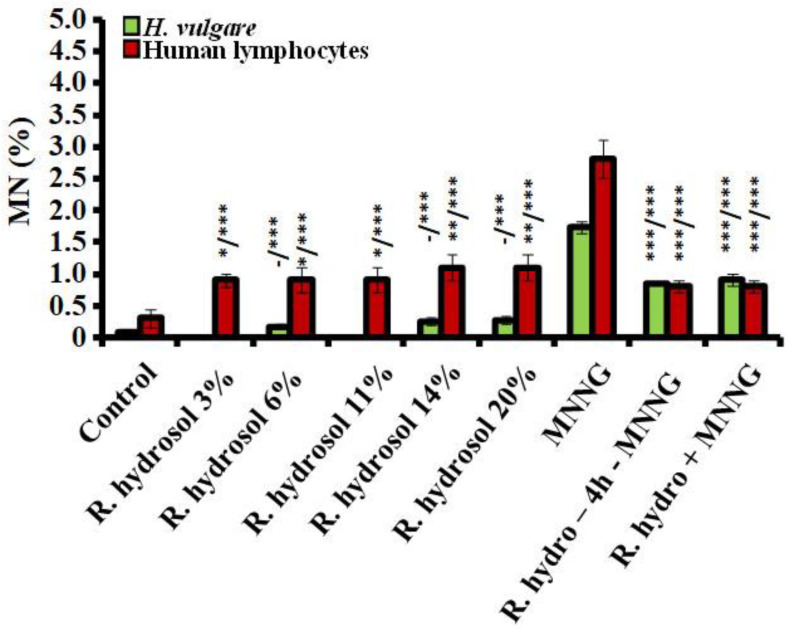
Frequency of MN observed after (i) treatment with different concentrations of *R. damascena* hydrosol and (ii) applying Schemes 1 and 2 with combined treatment with hydrosol and MNNG (50 μg/mL) in *H. vulgare* and in human lymphocytes in vitro (- n.s, * *p* < 0.05, ** *p* < 0,01, *** *p* < 0.001; before slash: compared with the negative control; after slash: compared with MNNG).

Using micronuclei (MN) as another genotoxicity end point, *R. damascena* hydrosol was found to have a weak genotoxic effect in barley. All tested concentrations induced very low frequencies of MN (from 0.17% ± 0.04 (for 6%) to 0.27% ± 0.06 (for 20%)) without any statistical differences relative to the negative control (0.08% ± 0.03) (Figure 8). Human lymphocytes were more sensitive to the hydrosol (*p* < 0.05, *p* < 0.01) than barley, as assessed by induction of MN. The micronuclei observed after the treatments ranged from 0.9% ± 0.10 (for 3%) to 1.1% ± 0.20 (for 14% and 20%). There was no established difference between the frequencies of MN induced by the tested concentrations. The genotoxic effect of the hydrosol assessed by MN was lower (*p* < 0.001) than that of MNNG, with frequencies in barley of 1.74% ± 0.09 and in lymphocyte cells of 2.82% ± 0.30 (Figure 8).

The genoprotective activity of the rose hydrosol against MNNG was well-expressed using MN as an end point and applying both schemes with combined treatment. Statistically significantly lower frequencies of micronuclei were observed in plant and human lymphocyte test systems (Figure 8).

In barley meristems, conditioning treatment with *R. damascena* hydrosol followed by MNNG and a 4 h intertreatment time induced a frequency of MN 0.85% ± 0.05, which is more than twice as low as that registered in variants with MNNG only. ln lymphocyte cells, this value was reduced by more than threefold (0.8% ± 0.10) relative to that induced by the direct mutagen. A similar reduction in the micronuclei values was obtained after applying Scheme 2 without any time between treatments. In barley, MNi were 0.91% ± 0.09 and in lymphocytes 0.8% ± 0.10; these values are close to those of the single *R. damascena* hydrosol treatments (Figure 8).

## 4. Discussion

DNA is exposed to various DNA-damaging agents, which can cause serious changes in the hereditary material. To reduce DNA damage, the main activity of researchers is to test the protective potential of various products, especially those of natural origin. *R. damascena* Mill. hydrosol is one of the main essential oil distillation products; however, there are fewer studies on its biological activities than those of essential oil. This especially applies to its cytoprotective and genoprotective potential. Hence, studying rose hydrosol’s anticytotoxic and antigenotoxic effects was of interest to us. It was also essential to address the biosafety of the rose product.

To ensure the safe use of various extracts, hydrosols, and other plant products, their cytotoxic and genotoxic potential should be evaluated. Our results show that treatment with rose hydrosol (4 h) did not affect the mitotic activity and did not induce or enhanced only to a limited extent the value of chromosomal disturbances and micronuclei in plant cells. The slightly higher cytotoxic and genotoxic effect of rose hydrosol in human lymphocytes is probably due to the lack of cell wall in this type of cells, which makes it easier for various chemical compounds (such as flavonoids and terpenes) found in the hydrosol to pass through the lymphocyte’s membrane. They can then be oxidized by ROS and converted into prooxidants and induce DNA injuries, especially at high concentrations [38].

In our previous study, we obtained the chemical composition of *R. damascena* hydrosol, where oxygenated monoterpenes were the highest relative percentage, at 65.87%, followed by benzenoid compounds at 9.51% and aliphatic hydrocarbons at 9.11% [22] (see Figure 1). Heterocyclic monoterpenes, sesquiterpenes hydrocarbons, oxygenated sesquiterpenes, and triterpenes were present in low quantities (1.19% to 1.4%). The bioactive substances of many hydrosols, mainly phenolic compounds, exhibit antioxidant and antiradical scavenging activity [29,39]. Such compounds include geraniol, geranyl acetate [40,41,42], citronellol [43], and linalool [44], which are the main compounds of rose hydrosol. These compounds can interact with free radicals, which are associated with DNA via external binding and can suppress DNA damage. In our previous study, we observed well-expressed antioxidant and radical scavenging activity of the *R. damascena* hydrosol [22]. Some authors have reported that *R. damascena* hydrosol can improve cell membrane integrity using compounds with radical scavenging activities [45] and can decrease the toxic effects induced by organophosphate pesticide chlorpyrifos [46].

Here, we tested the cytoprotective and genoprotective potential of *R. damascena* hydrosol against MNNG, a widely used experimental genotoxin that induces DNA damage due to its alkylating capacity [47]. As a result, DNA double-strand breaks and inhibition of replication can occur.

The application of rose hydrosol decreased the cytotoxic effect of MNNG, as it significantly increased the mitotic activity and cell proliferation (assessed by two different endpoints) after combined treatment with two different experimental schemes in both test systems we used.

Our results show that *R. damascena* hydrosol can reduce the genotoxic effect of the mutagen. The genoprotectivity of the hydrosol was manifested in both plant and lymphocyte test systems. The antigenotoxic potential of the *R. damascena* hydrosol against MNNG is revealed as decreases in the frequencies of both chromosomal aberrations and micronuclei induced by genotoxin, irrespective of the experimental treatment scheme. A reduction in the yield of “aberration hot spots” was also detected. After MNNG treatment, three “aberration hot spots” were observed in segments located directly adjacent to the centromeres in heterochromatin-rich regions. These regions are highly condensed, gene-poor, and transcriptionally silent. Five additional aberration hot spots were detected in less condensed gene-rich terminal regions, whereas samples treated with *R. damascena* hydrosol varied from one to three aberration hot spots: one located in the heterochromatin-rich region and two in euchromatin regions. The same was observed in the combined treatment variants. Our previous studies showed [37,48] that the aberrations within the heterochromatin-containing segments seem to be more harmful for survival than those induced in gene-rich regions. For the studies presented here, this means that the “aberration hot spots” observed in the variants treated with *R. damascena* hydrosol, as well as in the treatment variants with hydrosol conditioning, were not only reduced in number but also located in chromosome areas where they were less dangerous.

The antigenotoxic potential of the rose hydrosol obtained in the present study is probably due to the presence of phenols and flavonoids, the main chemical compounds in our previous study on aromatic products [22]. When rose hydrosol is applied as a conditioning treatment with non-toxic or low-toxicity concentrations, these compounds probably express their antioxidant activity and interact with the genotoxin MNNG, preventing its DNA-damaging effect. The well-expressed cytoprotective and genoprotective effects of rose hydrosol proposed the activation of at least two cellular defense mechanisms. In addition, with the antioxidant action of various phenols and flavonoids present in the aromatic product that block the action of free radicals, a repair pathway for N-alkylated bases induced by MNNG, such as base excision reparation (BER) and nucleotide excision reparation (NER), could also be activated [49]. Similar well-pronounced anticytotoxic and antigenotoxic effects against the same alkylating mutagen MNNG were also observed in our previous studies for *R. alba* produced after essential oil distillation [50]. Chemical analysis of the *R. damascena* hydrosol used in the present study showed similar chemical compounds to that of this rose product but in a different quantitative proportion. Our previous investigation demonstrated the well-expressed defense potential of monoterpenoid geraniol against MNNG, which is one of the main rose oil chemical components, as well as the hydrosol we tested [41]. The protective potential of *R. damascena* hydrosol is likely realized in the same way as in this rose oil distillation product. Further research could contribute to a more detailed study of the protective mechanism of rose hydrosol against various DNA-damaging agents.

## 5. Conclusions

*R. damascena* Mill. hydrosol generated during water–steam distillation of essential oil was found to exhibit effective cyto- and genoprotective activity against the harmful effect of MNNG applied in combined treatments with the mutagen in two experimental test systems. The obtained data are promising for further applications of rose hydrosol as a natural product with pharmacological potential.

## Data Availability

All the obtained data of the research are presented in the manuscript.
